# Characteristics and Predictors of Venous Thromboembolism Among Lymphoma Patients Undergoing Chemotherapy: A Cohort Study in China

**DOI:** 10.3389/fphar.2022.901887

**Published:** 2022-05-23

**Authors:** Yue Chen, Haike Lei, Wei Wang, Jie Zhu, Chensi Zeng, Zhuo Lu, Luchun Li, Dairong Li, Bo Long, Haixia Liu

**Affiliations:** ^1^ Chongqing Key Laboratory of Translational Research for Cancer Metastasis and Individualized Treatment, Chongqing University Cancer Hospital, Chongqing, China; ^2^ Chongqing Cancer Multi-Omics Big Data Application Engineering Research Center, Chongqing University Cancer Hospital, Chongqing, China

**Keywords:** VTE, lymphoma patients, chemotherapy, prospective cohort, targeted thromboprophylaxis

## Abstract

**Background:** Venous thromboembolism (VTE) is a potential complication among lymphoma patients. We evaluated the incidence rate and predictors of VTE in lymphoma patients undergoing chemotherapy.

**Methods:** The present study retrospectively studied 1,069 patients with lymphoma who were treated with chemotherapy from 2018 to 2020. We investigated clinical predictors of VTE among all patients. The follow-up results were obtained *via* telephone communication and from inpatient and outpatient records.

**Results:** A total of 1,069 patients underwent chemotherapy for lymphoma. During a mean follow-up of 23.1 months, 52 (4.9%) patients developed VTE. According to a multivariate analysis, the five variables found to be independently associated with VTE were male sex (HR 2.273, 95% CI 1.197–4.316, *p* = 0.012), age >64-years-old (HR 2.256, 95% CI 1.017–5.005, *p* = 0.045), the number of cycles of chemotherapy (HR 4.579, 95% CI 1.173–17.883, *p* = 0.029), platelet count ≥350 × 10^9^/L (HR 2.533, 95% CI 1.187–5.406, *p* = 0.016), and D-dimer >0.5 mg/L (HR 4.367, 95% CI 2.124–8.981, *p* < 0.001).

**Conclusion:** This population-based study confirms the risk factors for VTE among patients with lymphoma who underwent chemotherapy and confirms that targeted thromboprophylaxis may reduce the burden of VTE in this population.

## Introduction

Venous thromboembolism (VTE), including deep vein thrombosis (DVT) and pulmonary embolism (PE), is a common complication of lymphoma, with an incidence of 1.5%–59.5% (Goldschmidt et al., 2003; Zhou et al., 2010). A prospective study on newly diagnosed lymphoma patients in Asia found a 1-year VTE incidence of 7.9% (Park et al., 2012). Another meta-analysis of 29 independent cohorts, including 18,018 patients and 1,149 events, found a VTE incidence rate of 5.3% in adult patients with lymphoma. Among these lymphoma patients, the incidence rate of VTE for patients with non-Hodgkin lymphoma (NHL) was 6.5%, which is significantly greater than that observed for patients with Hodgkin lymphoma (HL) (4.7%) (Caruso et al., 2010). Compared to patients with several other types of cancer, patients with lymphoma are at significantly higher risk for VTE, especially those undergoing chemotherapy, for whom the VTE incidence rate may reach 15% or higher (Caruso et al., 2010; Falanga et al., 2012).

The development of VTE is related to various factors, including patient characteristics (i.e., bedridden status, central venous catheter use, older age, and prior history of venous thrombosis), disease status (i.e., surgery, comorbidities, coagulation function, and tumor progression), and therapeutic effects (i.e., chemotherapy, targeted therapy, and immunotherapy) (Reitsma et al., 2012; Timp et al., 2013; Mahajan et al., 2019). These factors have a significant impact on quality of life, additional anticoagulant therapy, increased risk of bleeding, financial burden, and tumor treatment, and the most serious consequence is increased mortality. Therefore, it is important to evaluate the clinical characteristics and risk factors associated with VTE among patients with lymphoma, as VTE has a considerable impact on the patient’s condition.

In this retrospective study, we aimed to clarify the clinical and laboratory variables, disease status, and treatment approaches that increase VTE risk in patients with lymphoma.

## Patients and Methods

### Study Population

Patients with lymphoma who underwent chemotherapy at the Chongqing University Cancer Hospital from January 2018 to December 2020 were considered possible candidates for the present study. The diagnosis of lymphoma was confirmed by histopathology, and the diagnostic criteria were based on the WHO classification. The exclusion criteria were as follows: juvenile patients (<18-years-old), patients with VTE, patients with other infectious or hematological malignancies as complications, and patients who refused or were unable to participate in the study. The present study was performed according to the guidelines of the Declaration of Helsinki and was approved by the Ethics Committee of The Chongqing University Cancer Hospital. Written informed consent was obtained from all subjects.

### VTE Diagnosis

VTE was defined only as a primary event, with an objective diagnosis of DVT and/or PE being performed during chemotherapy. DVT (lower extremity thrombosis, upper extremity thrombosis, central venous catheter thrombosis, portal vein thrombosis) was confirmed by color and Doppler ultrasound examination or venogram. PE was confirmed by ventilation/perfusion scan and/or computed tomography angiography.

### Data Collection

General, diagnostic and treatment information was collected for all patients. The specific data collected included age, sex, body mass index (BMI), histological type, Ann Arbor stage, Eastern Cooperative Oncology Group (ECOG) performance status, central venous catheter blood cell count, coagulation index, chemotherapy regimens, number of cycles of chemotherapy, and thrombosis location. All patients were routinely followed up *via* telephone communication, outpatient visits or hospitalization information. Telephone follow-ups were performed every 6 months after discharge. Patients were followed from the time of diagnosis until the development of VTE, death, loss to follow-up, whichever came first.

### Statistical Analysis

All statistical analyses were performed using SPSS 26.0 software. Patient demographics and clinical characteristics were summarized with descriptive statistics. Count data were compared using the χ2 test. Normally distributed measurement data were compared using an independent sample *t* test, and nonnormally distributed measurement data were compared using a corrected *t* test. Univariate and multivariate Cox regression analyses were used to identify the predictors associated with VTE in patients with lymphoma who underwent chemotherapy. *p* < 0.05 was considered statistically significant.

## Results

A total of 1,069 patients (635 males, 59.5%) with a mean age of 55.6 ± 14.4 years were included in the analyses from January 2018 to December 2020. Hodgkin lymphoma was diagnosed in 106 patients (9.9%), B-cell lymphoma was diagnosed in 788 patients (73.7%), T-cell lymphoma was diagnosed in 87 patients (8.1%), and NK/T-cell lymphoma was diagnosed in 88 patients (8.2%). Most patients had a good ECOG performance status (≤ECOG grade 1: 83.7%), while approximately 64.3% of patients had Ann Arbor stage III/IV disease. Only 6.3% of all patients received a central venous catheter (CVC). The most common chemotherapy regimen used was R-CHOP (rituximab, cyclophosphamide, adriamycin, vincristine and prednisone) or CHOP. However, 92.4% of patients with HL received the ABVD (adriamycin, bleomycin, vinblastine and dacarbazine) regimen. Ninety percent of patients had only 1-5 cycles of chemotherapy (Table 1).

**TABLE 1:** Clinical characteristics of lymphoma patients with or without VTE.

Characteristics	Total (%)	VTE (%)	No VTE (%)	*p* Value
Subjects, n	1069 (100)	52 (4.9)	1017 (95.1)	
Males, n (%)	635 (59.4)	38 (73.1)	597 (58.7)	0.043
Age, (years)				0.000
≤45	228 (21.3)	9 (17.3)	219 (21.5)	
46–64	506 (47.3)	14 (26.9)	492 (48.4)	
>64	335 (31.4)	29 (55.8)	306 (30.1)	
BMI, (kg/m^2^)	23.3 ± 3.6	23.2 ± 3.2	23.3 ± 3.6	0.960
ECOG, n (%)				0.018
0	337 (31.5)	21 (40.4)	316 (31.1)	
1	558 (52.2)	18 (34.6)	540 (53.1)	
2	167 (15.6)	11 (6.6)	156 (15.3)	
3	7 (0.7)	2 (3.8)	5 (0.5)	
Ann Arbor, n (%)				
I	120 (11.2)	9 (17.3)	111 (10.9)	0.480
II	261 (24.4)	11 (21.2)	250 (24.6)	
III	228 (21.3)	8 (17.3)	219 (21.5)	
IV	460 (43)	23 (44.2)	437 (43)	
Central venous catheter, n (%)	67 (6.3)	3 (5.8)	64 (6.3)	0.879
White blood cell count (>11×10^9^/L), n (%)	974 (91.1)	48 (92.3)	926 (91.1)	0.750
Hemoglobin (<100 g/L), n (%)	190 (17.8)	15 (28.8)	175 (17.2)	0.032
Platelet count (≥350×10^9^/L), n (%)	101 (9.4)	9 (17.3)	93 (9)	0.047
D-dimer (>0.5 mg/L), n (%)	544 (50.9)	42 (80.8)	10 (19.2)	0.000
Histological type, n (%)				0.059
Hodgkin lymphoma	106 (9.9)	7 (13.5)	99 (9.7)	
B-cell lymphoma	788 (73.7)	32 (61.5)	756 (74.3)	
T-cell lymphoma	87 (8.1)	9 (17.3)	78 (7.7)	
NK/T-cell lymphoma	88 (8.2)	4 (7.7)	84 (8.3)	
Chemotherapy regimens, n (%)				0.160
ABVD	98 (9.2)	6 (11.5)	92 (9)	
CHOP/R-CHOP	590 (55.2)	26 (50)	564 (55.5)	
P-GEMOX	56 (5.2)	3 (5.8)	53 (5.2)	
DICE/R-DICE	30 (2.8)	3 (5.8)	27 (2.7)	
R-EPOCH	47 (4.4)	0	47 (4.6)	
GDP/R-GDP	51 (4.8)	6 (11.5)	45 (4.4)	
BR	7 (0.7)	0	7 (0.7)	
Others	19,017.8)	8 (15.4)	182 (17.9)	
Cycles of chemotherapy, n (%)				0.001
1–5	996 (93.2)	42 (80.8)	954 (93.8)	
6–10	52 (4.8)	7 (13.5)	45 (4.4)	
≥11	21 (2.0)	3 (5.7)	18 (1.8)	

BMI, body mass index; VTE, venous thromboembolism; ECOG, eastern cooperative oncology group; ABVD, adriamycin, bleomycin, vinblastine and dacarbazine; R-CHOP, rituximab, cyclophosphamide, doxorubicin, vincristine and prednisone; P-GEMOX, pegaspargase, gemcitabine and oxaliplatin; R-DICE, rituximab, cisplatin, ifosfamide, etoposide and dexamethasone; R-EPOCH, rituximab, etoposide phosphate, prednisone, vincristine sulfate, cyclophosphamide and doxorubicin hydrochloride; R-GDP, rituximab, gemcitabine, dexamethasone and cisplatin; BR, bendamustine and rituximab.

During a mean follow-up of 23.1 months, 52 (4.9%) patients were diagnosed with VTE, and all the patients had DVT. Hodgkin lymphoma and non-Hodgkin lymphoma accounted for 6.7 and 4.7% of VTE patients, respectively. These patients developed VTE while undergoing chemotherapy, with a median time to event of 3.45 months. All episodes of VTE occurred after chemotherapy treatment was initiated.

**FIGURE 1:**
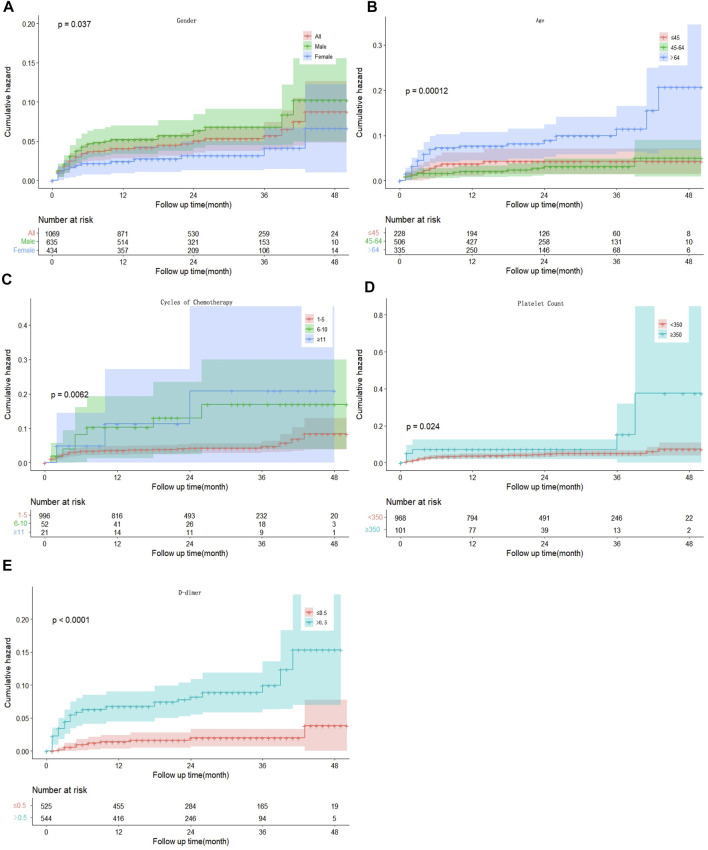
Kaplan–Meier curves for the cumulative incidence of VTE patients by sex **(A)**, age **(B)**, cycles of chemotherapy **(C)**, platelet count **(D)** and D-dimer concentration **(E)**. VTE, venous thromboembolism.

Univariate competing risks regression demonstrated that male sex (hazard ratio [HR] 1.919, 95% confidence interval [CI] 1.039–3.542, *p* = 0.037), age >64-years-old (HR 2.438 95% CI 1.153–5.157, *p* = 0.020), 6–10 cycles of chemotherapy (HR 2.916, 95% CI 1.304–6.518, *p* = 0.009), platelet count ≥350 × 10^9^/L (HR 2.229, 95% CI 1.085–4.580, *p* = 0.029), and D-dimer >0.5 mg/L (HR 4.593, 95% CI 2.229–9.176, *p* < 0.001) were associated with an increased risk of developing VTE. Body mass index, ECOG grade, Ann Arbor stage, CVC, white blood cell count, hemoglobin count and chemotherapy regimen were not associated with VTE development. To exclude the influence of confounding factors, all possible risk factors were added to the multivariate model. Conclusively, male sex (HR 2.273, 95% CI 1.197–4.316, *p* = 0.012), age >64-years-old (HR 2.256, 95% CI 1.017–5.005, *p* = 0.045), number of cycles of chemotherapy (HR 4.579, 95% CI 1.173–17.883, *p* = 0.029), platelet count ≥350 × 10^9^/L (HR 2.533, 95% CI 1.187–5.406, *p* = 0.016), and D-dimer >0.5 mg/L (HR 4.367, 95% CI 2.124–8.981, *p* < 0.001) were still statistically significant (Table 2; Figure 1).

**TABLE 2:** HRs (95% confidence intervals) for risk factors associated with venous thromboembolism.

Variables	Univariate		Multivariate^a^	
	HR (95%CI)	P	HR (95%CI)	P
Male sex	1.919 (1.039–3.542)	0.037	2.273 (1.197–4.316)	0.012
Age				
≤45	1 (ref.)			
46–64	0.730 (0.316–1.687)	0.461	0.769 (0.323–1.830)	0.553
>64	2.438 (1.153–5.157)	0.020	2.256 (1.017–5.005)	0.045
Cycles of chemotherapy				
1–5	1 (ref.)			
6–10	2.916 (1.304–6.518)	0.009	3.790 (1.566–9.171)	0.003
≥11	3.129 (0.965–10.141)	0.057	4.579 (1.173–17.883)	0.029
Platelet count ≥350 × 109/L	2.229 (1.085–4.580)	0.029	2.533 (1.187–5.406)	0.016
D-dimer >0.5 mg/L	4.593 (2.229–9.176)	<0.001	4.367 (2.124–8.981)	<0.001

a Adjusted for age, sex, BMI, ECOG, ann arbor, central venous catheter, white blood cell count, hemoglobin, platelet count, D-dimer, histological type, chemotherapy regimens, and cycles of chemotherapy.

## Discussion

The incidence of and risk factors for VTE among adult patients with lymphoma were investigated in the present study. A VTE incidence of 4.9% supported the similar prevalence (4%) found in another retrospective study, which included the second-largest population of lymphoma patients analyzed for VTE risk in California (Mahajan et al., 2014). The rate of VTE was 6.7% in patients with HL and 4.7% in patients with NHL, differing from a previous study (Caruso et al., 2010). A possible reason for the difference was that our research focused on patients who underwent chemotherapy and excluded many NHL patients undergoing other types of treatment, such as surgery and targeted therapies. Another reason may be related to regional differences.

In terms of individual risk factors, we reported male sex, age >64-years-old, increased cycles of chemotherapy, platelet count ≥350 × 10^9^/L and D-dimer >0.5 mg/L as potential risk factors for developing VTE. This finding was in accordance with a previous study that identified female sex, older age, ECOG performance scores ≥2 and anemia (hemoglobin <100 g/L) as predictors of VTE (Byun et al., 2019). A previous study also suggested that patients with platelet abnormalities prior to undergoing chemotherapy and patients at Ann Arbor stage III/IV were associated with a significantly higher risk of developing VTE (Li et al., 2021).

Several previous studies suggested that older age is a risk factor for cancer-associated VTE. Park et al. identified age >60 years as a risk factor for VTE in patients with diffuse large B-cell lymphoma (Park et al., 2012). Another study from Asia also reported a similar conclusion in primary central nervous system lymphoma patients who underwent chemotherapy (Byun et al., 2019). A recent analysis, which included 16,755 patients with NHL, confirmed that those who were aged ≥45 years and above had an increased risk of VTE (Mahajan et al., 2014). In the present study, we discovered a positive relationship between age >64-years-old and VTE, which is consistent with the aforementioned studies.

Male sex might be a potential risk factor for VTE in patients with lymphoma in the present study. Our research showed a 2.27-fold higher risk of VTE in male patients than in female patients. However, a study including 304 lymphoma patients who received chemotherapy investigated the risk of VTE occurring in women and found that it was significantly higher than that in other patients. The results of another study that included seven academic centers in Korea also suggested that female sex was independently associated with VTE in patients with primary central nervous system lymphoma (Byun et al., 2019). Although these results were inconsistent with our study, the data are far from conclusive. Several other studies did not find an association between female sex and the risk for VTE (Sanfilippo et al., 2016; Rupa-Matysek et al., 2017; Hohaus et al., 2018; Rupa-Matysek et al., 2018).

A platelet count ≥350 × 10^9^/L is a component of the Khorana Risk score for VTE development in cancer patients and is considered to increase the risk of VTE (Khorana et al., 2008). Li et al. suggested that an abnormal platelet count (<125 or >350 × 10^9^/L) increased the risk of VTE by more than 60 times (Li et al., 2021). Platelet abnormalities are an independent risk factor for VTE in lymphoma patients, but little is known about the pathophysiological mechanism underlying this relationship. This might be related to the expression and release of some cytokines by tumor cells, leading to the activation of platelet and coagulation pathways. Additionally, tumor cells can secrete some inflammatory cytokines, such as tumor necrosis factor α and interleukin-β, which can result in platelet activation and the expression of a procoagulant phenotype by endothelial cells (Falanga et al., 2009).

In our study, we found that increasing the number of cycles of chemotherapy led to a higher risk of VTE in lymphoma patients. The incidence of VTE in patients who underwent more than 10 cycles of chemotherapy was significantly higher than that in patients who underwent 1-5 cycles of chemotherapy. The results supported findings from several studies showing a relationship between chemotherapy and the risk of VTE (Horsted et al., 2012; Sanfilippo et al., 2016). A prior study including 2,650 patients who underwent orchiectomy for testicular cancer also suggested that an increasing number of chemotherapy cycles was an independent risk factor for VTE (Robinson et al., 2020). Many chemotherapeutic drugs are known to be associated with VTE. The increasing number of cycles of chemotherapy leading to the development of VTE may be related to drug accumulation effects.

D-dimer is a typical marker of VTE and is widely used in clinical practice. Several studies confirmed a correlation between D-dimer levels and VTE in hematologic malignancies (Ay et al., 2009; Libourel et al., 2016). Libourel et al. showed that the risk of VTE among patients with D-dimer >4.0 mg/L was 32 times higher than that among patients with D-dimer ≤0.5 mg/L (Libourel et al., 2016). Another study of 111 patients from the Vienna Cancer and Thrombosis Study (CATS) with hematologic malignancies (lymphoma and multiple myeloma) demonstrated that elevated D-dimer levels (>1.4 mg/L) were associated with an increased risk of VTE (Ay et al., 2009). Notwithstanding, data to support a cutoff for increased D-dimer levels in routine clinical decision-making are currently lacking. However, there is a well-established trend that increasing D-dimer levels lead to an increased risk of thrombosis.

The current research was a single-center retrospective study. We confirmed the risk factors for VTE among patients with lymphoma who underwent chemotherapy. Compared to other risk models (i.e., the Khorana score, the ONKOTEV score and the TiC-Onco score), age, sex, and the number of cycles of chemotherapy were included as predictors. Although the study had a small sample size from a single center, these variables were more available clinically, which provided a reference for future research. The limitations of this study are related to regional bias and the small sample size. Another limitation was the possible omission of asymptomatic VTE due to this follow-up method. Since it was a retrospective study, some variables, such as VTE history and hospitalization status, were not available, which might affect the results. The strength of our study was an adequate follow-up. Multicenter, large-scale cohorts with more statistical power are needed to validate the findings of the present study, which will help to verify effective predictors and implement early intervention to improve the quality of life and prolong the survival of patients.

## Conclusion

In this paper, we demonstrated that male sex, older age, an increased number of cycles of chemotherapy, a platelet count ≥350×10^9^/L and a D-dimer >0.5 mg/L were associated with VTE incidence in lymphoma patients. Early VTE identification and intervention are critically important for clinical practice**.**

## Data Availability

The original contributions presented in the study are included in the article/supplementary materials, further inquiries can be directed to the corresponding authors.
